# Effects of head motion on postural stability in healthy young adults with chronic motion sensitivity

**DOI:** 10.1186/s40945-020-00077-9

**Published:** 2020-03-30

**Authors:** Abdulaziz A. Albalwi, Eric G. Johnson, Ahmad A. Alharbi, Noha S. Daher, Tim K. Cordett, Oluwaseun I. Ambode, Fahad H. Alshehri

**Affiliations:** 1 Department of Physical Therapy, Faculty of Applied Medical Sciences, Tabuk University, Duba Road, Tabuk, 71491 Saudi Arabia; 2 Department of Physical Therapy, School of Allied Health Professions, Loma Linda University, Loma Linda, CA USA; 3 Department of Allied Health Studies, School of Allied Health Professions, Loma Linda University, Loma Linda, CA USA

**Keywords:** Motion sensitivity, Postural stability, Equilibrium, Head motion

## Abstract

**Background:**

Motion sensitivity, or motion sickness, is common in modern vehicular and visually stimulating environments. Several studies have shown a relationship between motion sensitivity and decreased postural stability. We aimed to evaluate the effects of head motion (horizontal and vertical) on postural stability in healthy adults with and without chronic motion sensitivity (CMS).

**Methods:**

Sixty healthy adult men and women (age, 20–40 years) with CMS (CMS group, *n* = 30) and without CMS (non-CMS group, *n* = 30) participated in the study. Postural stability was assessed during three conditions (static, horizontal head motion, and vertical head motion) using computerized dynamic posturography. Group and condition-related differences in equilibrium scores were evaluated.

**Results:**

There was no significant group x condition interaction (F_2,114_ = 0.9, partial ƞ^2^ = 0.04, *p* = 0.35). However, significant condition-related differences in equilibrium scores were observed (F_2,114_ = 26.4, partial ƞ^2^ = 0.31, *p* < 0.001). Equilibrium scores were significantly worse in the horizontal and vertical head motion conditions compared to those in the static condition (*p* < 0.001), but were comparable in vertical and horizontal head motion conditions (*p* = 0.27).

**Conclusions:**

Postural stability was lower in the horizontal and vertical conditions compared to the static condition. However, horizontal and vertical head motions had comparable effects on postural stability in both CMS and non-CMS groups, contrary to our expectations.

## Background

Motion sensitivity, or motion sickness, is common in modern vehicular and visually stimulating environments, and individuals with normal vestibular function are susceptible [1]. It has been reported that 28.4% of the population experience motion sensitivity [2], and it is more common in women than in men [2, 3]. Transportation, such as cars, trains, amusement park rides, airplanes, boats, and entertainment innovations (e.g., virtual reality), play a major role in increasing the prevalence of motion sensitivity [4]. Furthermore, transportation, in general, is a part of everyday life for most people [5].

Motion sensitivity is traditionally defined as the onset of nausea or vomiting experienced by individuals traveling by air, sea, space, and land, leading to impaired function [6]. Symptoms of motion sensitivity include visual and postural instability, pallor, sweating, excess salivation, headaches, drowsiness, malaise, nausea, and vomiting [5, 7]. The most widely accepted mechanism is the sensory conflict theory [8], which states that motion sensitivity is the result of a sensory input mismatch (visual, vestibular, and proprioceptive) [6, 9, 10]. In other words, information provided by one sensory system does not match the expected input from another system; usually, a mismatch between the vestibular and visual systems is involved [6, 9, 10]. An alternative theory is the postural instability theory, which states that motion sensitivity is caused by a loss of postural control [11].

Postural stability is a complex task, requiring the proper integration of sensory inputs from visual, vestibular, and proprioceptive systems [12–14]. Therefore, postural stability includes the coordination of movement strategies to maintain the center of body mass during both self-initiated and externally triggered disturbances in balance [14]. Individuals with motion sensitivity often complain of postural instability and/or dizziness, which is a bothersome feeling that can be associated with head motion and not necessarily the result of vestibular dysfunction [15, 16]. Stimulation of the vestibular system activates the vestibulo-ocular reflex and the vestibulospinal reflex, while stimulation of the upper neck-joint receptors activates the cervico-ocular reflex [17]. Consequently, both head and neck rotation contribute to stimulating these reflexes [18]. In addition, increased postural instability can be stimulated by either active head rotation or head tilt in patients with vestibular dysfunction [19, 20] as well as in healthy individuals [21, 22]. Finally, several studies have shown a relationship between motion sensitivity and postural instability [23–27]. For example, Owen et al. [27] found that greater postural instability was correlated with motion sensitivity. The authors reported that motion sensitivity susceptibility correlated most strongly with postural instability during conditions of visual and somatosensory feedback was absent or distorted.

Additional research supports an effect of head movement on postural stability. For example, Guedry and Benson [28] investigated Coriolis cross-coupling effects on healthy individuals and found that head movements can cause nausea and disorientation. Furthermore, head movements in weightlessness, especially in the pitch direction, are likely to cause motion sensitivity [29]. However, horizontal movements are more likely relevant to the routine activities of daily life and comprise a substantial portion of the head movements associated with daily balance activities [19]. Lackner and Graybiel [30] examined the effects of head movement direction (i.e., yaw, roll, and pitch) on motion sensitivity and found that all movements provoke sensitivity. In addition, Paloski et al. [21] examined the effects of different head movement frequencies on postural control in healthy individuals and found that postural instability was increased during dynamic head tilts. Thus, postural stability may be worse during vertical head motion compared to that during horizontal head motion.

Given the functional relationship between dynamic head motion and postural stability, people with chronic motion sensitivity (CMS) may experience additional challenges in their daily lives. To our knowledge, the effects of head motion on postural stability in individuals with CMS have not been reported. Therefore, we aimed to investigate the effects of head motion (horizontal, vertical) on postural stability in healthy adults with and without CMS. The primary hypothesis was that postural stability during head motion would be worse in the CMS group compared to that in the non-CMS group. The secondary hypothesis was that postural stability would be worse during vertical head motion compared to that during horizontal head motion for adults with or without CMS.

## Methods

### Design

The present study utilized an observational cross-sectional design.

### Participants

A total of 60 young adult participants aged from 20 to 40 years old from Loma Linda University and the local community (30 men and 30 women with a mean age of 26.8 ± 4.3 years and a body mass index [BMI] of 24.9 ± 4.6 kg/m^2^) were recruited for this study via email, word of mouth, and flyers posted around the university campus. Participants with a history of vestibular disorders, neurological pathology, head or cervical trauma, lack of a normal cervical spine active range of motion, Motion Sensitivity Susceptibility Questionnaire-Short Form (MSSQ-SF) [31] score between the 25th and 30th percentile, and those who were taking any medications that might affect balance were excluded from the study. Participants were divided into those with a self-reported history of CMS and an MSSQ-SF score in the 30th percentile or more (CMS group, *n* = 30) and those without a self-reported history of CMS and a MSSQ-SF score in the 25th percentile or less (non-CMS group, *n* = 30). The MSSQ-SF does not have a specific cut-off value; thus, the use of the 30th percentile was based on the recommendation of the author of the MSSQ-SF and the findings of a previous study that reported the 30th percentile as the lowest score in the CMS group [32]. We excluded participants who scored between the 25th and 30th percentile to create a “gap” between the two groups. This study was conducted at Loma Linda University in the Physical Therapy Neuroscience Research Laboratory.

### Ethics

All participants provided written informed consent, and the study was approved by the University Institutional Review Board.

### Procedures

All participants completed the MSSQ-SF, which was designed to assess the types of motion that cause motion sickness in children and adults [31]. The MSSQ-SF has a high correlation with the MSSQ–Long Form (*r* = 0.93). In addition, the MSSQ-SF exhibits high internal consistency (Cronbach’s alpha = 0.87), test–retest reliability (*r* = 0.9), and a significant correlation between Section A (Child) and Section B (Adult) results (*r* = 0.68) [31].

In addition, participants self-reported their physical activity level and anthropometric measurements (weight and height) were taken. Participants were then trained on the specific parameters of cervical rotation, flexion, and extension. To prevent falling, participants donned a safety harness and two investigators stood behind the participant during all postural stability testing. Postural stability was measured during three conditions (static, horizontal head motion, and vertical head motion) using a computerized dynamic posturography (CDP) forceplate system (Bertec Balance Advantage Dynamic CDP, Bertec Corporation, Columbus, OH, USA). Each condition included three twenty-second trials; the mean of the three trials was analyzed.

Participants performed all conditions while standing on a CDP force plate with bare feet. For dynamic conditions (head motions), the head velocity and amplitude was selected based on slow-speed walking, as this is a common physical activity of daily living for most individuals [33]. Based on the reported normal head velocity and amplitude during walking, a velocity of 1.5 Hz [34], and head motion amplitudes of 11 degrees in the horizontal direction and 8 degrees in the vertical direction [35] were utilized. The dynamic conditions were measured with the participants performing active head motions (horizontal or vertical), in randomized order, moving their heads to the auditory cue of a metronome set at 1.5 Hz.

**Fig. 1:**
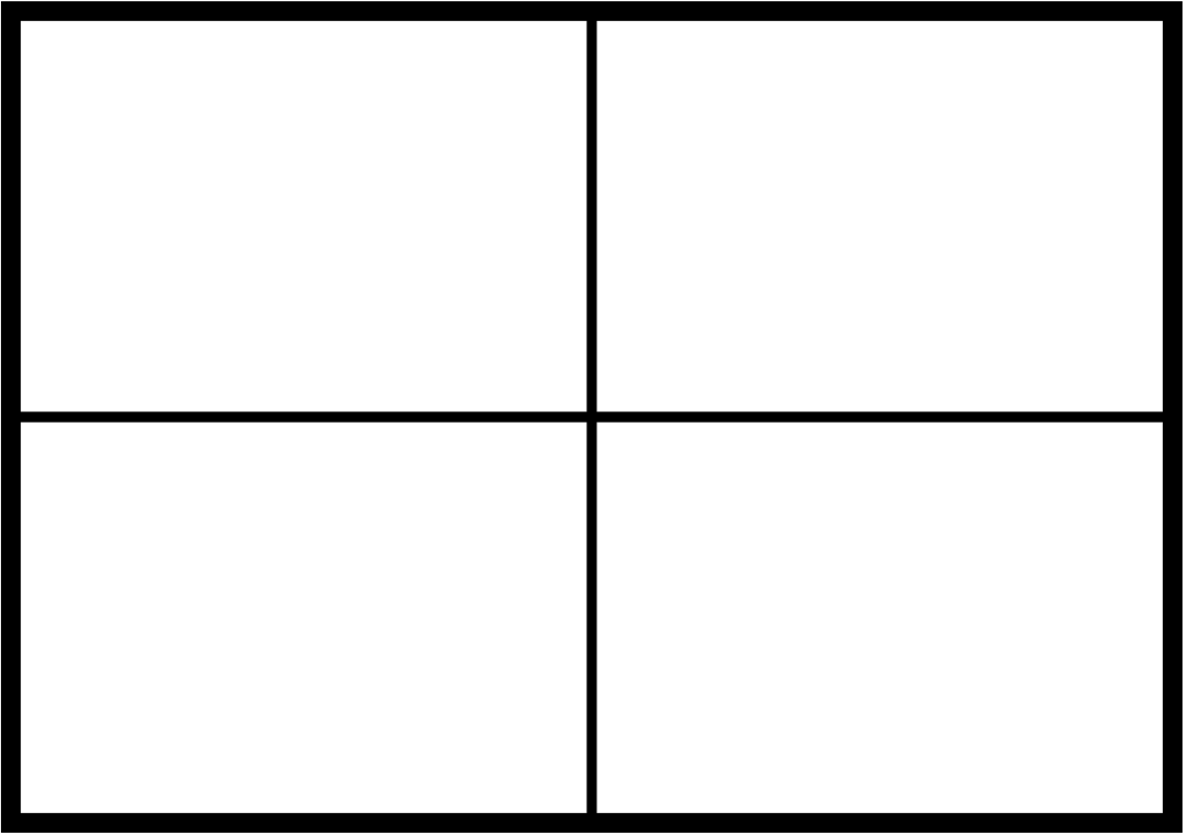
Grid to guide the amplitude of horizontal (11°) and vertical (8°) head motions

In contrast to several previous studies, in which participants performed head movements with their eyes closed during sensory organization testing by holding their hands 15 degrees to each side of their face to control the range of motion [19, 20, 36], the present study utilized a head-mounted laser pointer (SenMoCOR LED/Laser, Orthopedic Physical Therapy Products, USA). Participants were instructed to keep their eyes open to guide the range of motion amplitude, which allowed the use of the amplitude and velocity of head motion during slow walking as a reference. Furthermore, people usually keep their eyes open during locomotion to explore the environment around them. Additionally, a grid was positioned at the same height as the participant’s eyes, 90 cm from the participant’s forehead, to aid in tracking the laser (Fig. 1). Under the guidance of the laser pointer and verbal cues from the investigators, the participants maintained a range of motion amplitude of approximately 11 degrees in the horizontal plane (5.5 degrees to each side) and 8 degrees in the vertical plane (4 degrees up and 4 degrees down).

Participants postural stability, or equilibrium score, was calculated using the following formula: Equilibrium Score (ES) = (12.5-(Max–AP COG Angle))/12.5 X 100.

The equation for sway angle is: Sway Angle CDP = arcsin (COGy/(.55*h)) where y = anterior-posterior sway axis and h = the subject’s height in [cm or inches]. The inverse Sin of the center of gravity was divided by 55% of the subject’s height. Subjects exhibiting little sway achieve equilibrium scores near 100, while subjects whose sway approaches their limits of stability achieve scores near zero. The equilibrium score ranges between 0 and 100%; higher values reflect better postural stability [37].

### Statistical analysis

A sample size of 60 participants was estimated using GPower software (version 3.1.2, University of Dusseldorf, Dusseldorf, Germany) assuming a medium effect size of (f = 0.25), a power of 0.80, and a level of significance (α) of 0.05. Data analyses were performed using the SPSS statistical package for Windows, version 22.0 (SPSS, Inc., Chicago, IL). Descriptive statistics are given as mean and standard deviation for quantitative variables, and as frequency and percentage (%) for categorical variables. Group differences in the frequency distribution of sex and physical activity level were evaluated using chi-square tests. Normality was assessed using the Kolmogorov-Smirnov test. Group differences in height, weight, and body mass index (BMI) were evaluated using the independent-sample *t*-test. Since significant group differences in age were observed, group and condition-related changes in equilibrium scores (static vs. horizontal vs. vertical) were examined using a mixed factorial analysis of variance (ANOVA), after controlling for age. Post hoc analyses with Bonferroni correction were conducted. The level of significance was set at *p* ≤ 0.05.

## Results

The 60 participants had a mean age of 26.8 ± 4.3 years and mean body mass index (BMI) of 24.9 ± 4.6 (kg/m^2^). There were no significant group differences in mean height (m), weight (kg), or BMI (kg/m^2^) (*p* > 0.05, Table 1). In addition, the groups did not differ in sex distribution or physical activity level (Table 1). However, there was a significant difference in mean age between the two groups (27.9 ± 4.5 vs. 25.6 ± 3.8, *p* = 0.04, Table 1). In addition, there was no significant difference in mean equilibrium score during static condition by group (93.8 ± 2.7 vs. 94.9 ± 1.3, *p* = 0.25).

**Table 1:** General baseline characteristics of the study participants (*N* = 60)

Characteristic	CMS (*n*_1_ = 30)	Non-CMS (*n*_2_ = 30)	*p* –value
Gender; n (%)			0.22
Female	13 (43.3)	17 (56.7)	
Male	17 (56.7)	13 (43.3)	
Age (years)	27.9 (4.5)	25.6 (3.8)	0.04
Height (m)	1.7 (0.1)	1.7 (0.1)	0.62
Weight (kg)	75.1 (20.6)	68.7 (14.6)	0.17
BMI (kg/m^2^)	25.8 (5.6)	24.1 (3.2)	0.14
Physical Activity; n (%)			0.29
Often	11 (36.7)	14 (46.7)	
Sometimes	16 (53.3)	15 (50.0)	
Never	3 (10.0)	1 (3.3)	

**Abbreviations:***SD* Standard Deviation; *CMS* Chronic Motion Sensitivity; *BMI* Body Mass Index

Results of the mixed factorial ANOVA are displayed in Table 2. There was no significant group by condition interaction (F_2,114_ = 0.9, partial ƞ^2^ = 0.04, *p* = 0.35). However, the mean equilibrium score differed significantly among the three tested conditions (F_2,114_ = 26.4, partial ƞ^2^ = 0.31, *p* < 0.001). Post hoc comparisons using Bonferroni adjustment revealed that mean ± standard error equilibrium scores were significantly different between horizontal head motion and static condition (92.4 ± 0.4 vs. 94.4 ± 0.3, *p* < 0.001) and vertical head motion condition compared to the static condition (91.9 ± 0.5 vs. 94.4 ± 0.3, *p* < 0.001), however, there was no significant difference between horizontal and vertical conditions (*p* = 0.27). Similar results were obtained after adjusting for age.

**Table 2:** Mean (SE) equilibrium scores during head motion by group (*N* = 60)

Condition^a^	CMS (*n*_1_ = 30)	Non-CMS (*n*_2_ = 30)	Group x condition p-value^*^ (partial ƞ^2^)
Static condition	93.8 (0.3)	94.9 (0.4)	0.11 (0.04)
Horizontal	91.1 (0.6)	93.6 (0.6)	
Vertical	90.7 (0.7)	93.1 (0.7)	

**Abbreviations:***SE* Standard Error; *CMS* Chronic Motion Sensitivity

^a^Significant difference between static and horizontal, and static and vertical conditions (*p* < 0.001)

^*^Mixed factorial ANOVA

## Discussion

Building upon the work of Owen et al. [27], the present study describes the effects of head motion on postural stability in healthy adults with and without CMS. The results demonstrate that postural stability differs by the condition of head motion. Postural stability was lower in the horizontal and vertical conditions compared to the static condition. However, horizontal and vertical head motions had comparable effects on postural stability in both CMS and non-CMS individuals, contrary to our expectations. Consistent with the present results, Mitsutake et al. [38] reported that postural stability during active horizontal head motion (during eyes open and closed conditions) was significantly decreased in stroke patients compared to that in healthy people. In addition, Paloski et al. [21] reported that healthy subjects were able to maintain an upright stance during static head tilts with eyes closed; however, postural stability was decreased during dynamic head tilts with eyes closed, especially with higher degrees of head tilt.

Many studies have measured unperturbed body sway before participants were exposed to visual motion stimuli [23–26]. The results from these studies demonstrate that pre-exposure postural stability is diminished in participants who became sick after motion exposure compared to that in those who did not become motion sick, consistent with the present results. Furthermore, a study by Stoffregen, Chen, and Koslucher [39] found that movement of the head and torso differed between participants who later became motion sick and those who did not. Together, these studies provide support for the postural instability theory. Owen et al. [27] reported that greater postural instability was correlated with motion sensitivity during conditions of visual and somatosensory feedback absence or distortion. The results of the present study do not support these assumptions; however, the conditions of postural stability in this investigation did not remove or distort visual or somatosensory input.

Sensory systems (visual, somatosensory, and vestibular), central processing, musculoskeletal systems, and neural pathways are essential for postural stability [40, 41]. To maintain postural stability, the vestibular system provides the central nervous system with information about head motion relative to space [42]. This information estimates the orientation of an individual in space and the degree of tilt from gravity vertical, which assists the individual in maintaining upright while standing and walking [42]. Paillard et al. [43] indicated that the vestibular system might be involved in CMS. Vestibular system involvement in CMS, and the stimulation of the vestibular system during head motion, may explain the observed reduced postural stability during head motion in the CMS group compared to that in the non-CMS group.

We also examined whether horizontal and vertical head movements differentially impact postural stability in adults with and without CMS. The results suggest that postural stability is similar in the CMS group compared to that in the non-CMS group for both horizontal and vertical head movements during conditions of normal vision and slow velocity head motion. Functional implications of horizontal and vertical head motions include a variety of standing activities (e.g., checking for traffic before crossing the street, looking in kitchen cabinets, and showering).

Horizontal movements are likely more relevant to routine activities of daily life and comprise a fundamental portion of head movements associated with daily balance activities [19]. In addition, horizontal eye and head motions are often utilized to guide changes in walking directions [44]. Consequently, we hypothesized that postural stability would be worse during vertical head motion compared to that during horizontal head motion within both groups; however, this hypothesis was not supported. Lackner and Graybiel [30] demonstrated that all movements (yaw, roll, and pitch) provoked motion sensitivity; however, pitch head movements (vertical motion) were the most stressful. In other investigations, individuals with vestibular disorders reported more falls while walking with vertical head movement than while walking with horizontal head movement [45]. However, others have reported that no difference exists in walking speed during horizontal and vertical head movements [46]. In the present study, the amplitude of the horizontal head motion was greater compared to that for the vertical head motion. Thus, we speculate that the difference in the amplitude of the head range of motions (11 degrees horizontal versus 8 degrees vertical) may explain the lack of significant differences between the head motion conditions.

Although the design of the present study included potential confounders (i.e., the visual cues during the laser-guided head motion and auditory attention to the metronome guiding head motion velocity), a previous study indicated that a secondary task, such as an auditory signal, does not affect balance control [21]. In addition, the performance of simultaneous tasks, such as walking while talking with friends and watching the world around us, is necessary during daily activities. Therefore, motor and cognitive tasks do not always require conscious attention and can be performed automatically [47]. Further study is needed to investigate the effects of dual-task performance on postural stability in individuals with CMS.

### Limitations

The present study had several limitations. The main limitation concerns the narrow age range of the participants (20–40 years of age); thus, the findings may not be generalizable to older adults. Also, a valid and reliable physical activity questionnaire was not utilized, and inactivity can affect postural stability [48]. Finally, vestibular function testing was not performed. Future studies should include additional age ranges, perform vestibular function testing, consider varying the head motion velocity and amplitude, and investigate whether standing gaze-stability exercises [32, 49] can improve postural stability.

## Conclusions

The results demonstrate that postural stability differs by the condition of head motion. Postural stability was lower in the horizontal and vertical conditions compared to the static condition. However, horizontal and vertical head motions had comparable effects on postural stability in both CMS and non-CMS groups, contrary to our expectations.

## Data Availability

The datasets used and/or analysed during the current study are available from the corresponding author on reasonable request.

## Abbreviations

ANOVA: Analysis of variance
BMI: Body mass index
CDP: Computerized dynamic posturography
CMS: Chronic motion sensitivity
MSSQ-SF: Motion Sensitivity Susceptibility Questionnaire-Short Form
SD: Standard Deviation
SE: Standard Error
